# Multiple Sclerosis (MS) as a demyelinating disease: a review on neurosurgical approaches

**Published:** 2012-11

**Authors:** Bahram Amin-Mansour

**Affiliations:** ^a^Department of Neurosurgery, Esfahan School of Medical Sciences, Esfahan, Iran.

## Abstract

**Background:**

Although the demyelinating disease is not among the routine neurosurgery practices, it has been creating serious challenges for neurosurgeons both in diagnostic and surgical procedures for a long time.

**Case Study:**

Pathologically, they are similar to grade II glioma– oligodendroglioma or mixed gliomas. In the neurosurgical context, they are large and solitary lesion mimicking metastasis – lymphoma and brain abscesses. Because of these characteristics, medical literatures show that several cases of demyelinating disease have been surgically treated.

**Diagnosis and Treatment approach:**

For an efficient differential diagnosis of demyelinating disease using neuroimaging techniques such as magnetic resonance imaging (MRI) different types of information should be available such as the age of patient, previous medical history as well as physical examination. T2- hyperintensity of demyelinating plaques – ovoid lesions with main axes perpendicular to corpus callosum (called Dawson’s fingers) and (open ring shaped) an incomplete peripheral enhancing with the gap facing towards the brain surface all are imaging signs of the presence of demyelinating disease.

Furthermore, in spinal cord any expanding lesion even with syrinx should be considered in differential diagnosis of Multiple Sclerosis (MS), particularly in aggressive demyelinating diseases common in Asian countries. All of the above mentioned points are discussed in detail in this study.

**Keywords:**

Multiple sclerosis, Demyelinating disease, Neurosurgery, Differential diagnosis


					Volume 4, Suppl. 1 Nov 2012
Publisher: Kermanshah University of Medical Sciences
URL: http://www.jivresearch.org
Copyright:
Congress of Iranian Neurosurgeons, 14-16 November 2012, Kermanshah, Iran
Paper No. 31
Multiple Sclerosis (MS) as a demyelinating disease: a review
on neurosurgical approaches
Bahram Amin-Mansour a,*
a
Department of Neurosurgery, Esfahan School of Medical Sciences, Esfahan, Iran.
Abstract:
Background: Although the demyelinating disease is not among the routine neurosurgery practices, it has been
creating serious challenges for neurosurgeons both in diagnostic and surgical procedures for a long time.
Case Study: Pathologically, they are similar to grade II glioma- oligodendroglioma or mixed gliomas. In the
neurosurgical context, they are large and solitary lesion mimicking metastasis - lymphoma and brain abscesses.
Because of these characteristics, medical literatures show that several cases of demyelinating disease have been
surgically treated.
Diagnosis and Treatment approach: For an efficient differential diagnosis of demyelinating disease using
neuroimaging techniques such as magnetic resonance imaging (MRI) different types of information should be
available such as the age of patient, previous medical history as well as physical examination. T2- hyperintensity of
demyelinating plaques - ovoid lesions with main axes perpendicular to corpus callosum (called Dawson's fingers)
and (open ring shaped) an incomplete peripheral enhancing with the gap facing towards the brain surface all are
imaging signs of the presence of demyelinating disease.
Furthermore, in spinal cord any expanding lesion even with syrinx should be considered in differential diagnosis of
Multiple Sclerosis (MS), particularly in aggressive demyelinating diseases common in Asian countries. All of the above
mentioned points are discussed in detail in this study.
Keywords:
Multiple sclerosis, Demyelinating disease, Neurosurgery, Differential diagnosis
* Corresponding Author at:
Bahram Amin-Mansour: Department of Neurosurgery, School of Medical Sciences, Esfahan, Iran. Tel: 09131184510,
Email: aminmansour@med.mui.ac.ir, (Amin-Mansour B.).

				

